# A computational method for design of connected catalytic networks in proteins

**DOI:** 10.1002/pro.3757

**Published:** 2019-11-19

**Authors:** Brian D. Weitzner, Yakov Kipnis, A. Gerard Daniel, Donald Hilvert, David Baker

**Affiliations:** ^1^ Department of Biochemistry University of Washington Seattle Washington; ^2^ Institute for Protein Design University of Washington Seattle Washington; ^3^ Howard Hughes Medical Institute University of Washington Seattle Washington; ^4^ Laboratory of Organic Chemistry ETH Zurich Zurich Switzerland

**Keywords:** biocatalysis, computational modeling, enzyme design, enzyme mechanism, protein design

## Abstract

Computational design of new active sites has generally proceeded by geometrically defining interactions between the reaction transition state(s) and surrounding side‐chain functional groups which maximize transition‐state stabilization, and then searching for sites in protein scaffolds where the specified side‐chain–transition‐state interactions can be realized. A limitation of this approach is that the interactions between the side chains themselves are not constrained. An extensive connected hydrogen bond network involving the catalytic residues was observed in a designed retroaldolase following directed evolution. Such connected networks could increase catalytic activity by preorganizing active site residues in catalytically competent orientations, and enabling concerted interactions between side chains during catalysis, for example, proton shuffling. We developed a method for designing active sites in which the catalytic side chains, in addition to making interactions with the transition state, are also involved in extensive hydrogen bond networks. Because of the added constraint of hydrogen‐bond connectivity between the catalytic side chains, to find solutions, a wider range of interactions between these side chains and the transition state must be considered. Our new method starts from a ChemDraw‐like two‐dimensional representation of the transition state with hydrogen‐bond donors, acceptors, and covalent interaction sites indicated, and all placements of side‐chain functional groups that make the indicated interactions with the transition state, and are fully connected in a single hydrogen‐bond network are systematically enumerated. The RosettaMatch method can then be used to identify realizations of these fully‐connected active sites in protein scaffolds. The method generates many fully‐connected active site solutions for a set of model reactions that are promising starting points for the design of fully‐preorganized enzyme catalysts.

1

The goal of *de novo* enzyme design is to create protein catalysts for any chemical reaction of interest.^1^, ^2^, ^3^, ^4^, ^5^ Several approaches have been developed to generate new enzyme active sites by searching for placements of catalytically competent side‐chain constellations in selected protein scaffolds or curated subsets of the Protein Data Bank containing up to several thousand protein structures.^6^, ^7^, ^8^, ^9^, ^10^, ^11^ Rosetta computational enzyme design calculations have proceeded by first generating an ideal active site, or theozyme, consisting of the reaction transition state surrounded by side‐chain functional groups positioned so as to maximize transition‐state stabilization. RosettaMatch is then used to search for geometrically compatible placements of these ideal active sites in protein scaffolds.^12^ While directed evolution has succeeded in maturing computational designs to have activities comparable to native enzymes,^13^, ^14^, ^15^, ^16^, ^17^, ^18^, ^19^ the activities of the original computational designs have generally been quite low. Achieving high catalytic activity directly from computation is an outstanding current challenge.

A route to increasing the activity of computational enzyme designs is suggested by the crystal structure of the optimized aldolase RA95.5‐8F which, with a *k*_cat_/*K*_m_ of ~33,800 ± 4,200 M^−1^ s^−1^, is 200,000‐fold more active than the original RA95.0 design (Figure 1a,b).^15^ In this structure (Figure 1c), the catalytic residues form an extensive hydrogen‐bond network (a catalytic quartet). In contrast, in the starting computational design, as in most such designs, there are only a small number of interactions between the designed catalytic residues. High catalytic residue connectivity has the advantage of allowing concerted transitions, such as proton shuffling, during catalysis and in preorganizing the active site residues in catalytically competent conformations. Indeed, highly‐connected catalytic side‐chain networks are frequently observed in native enzymes. A limitation of the RosettaMatch method, which focuses on the geometry of side‐chain interactions with the transition state, is that the extent of interaction of catalytic residues with each other cannot be directly specified.

**Figure 1:**
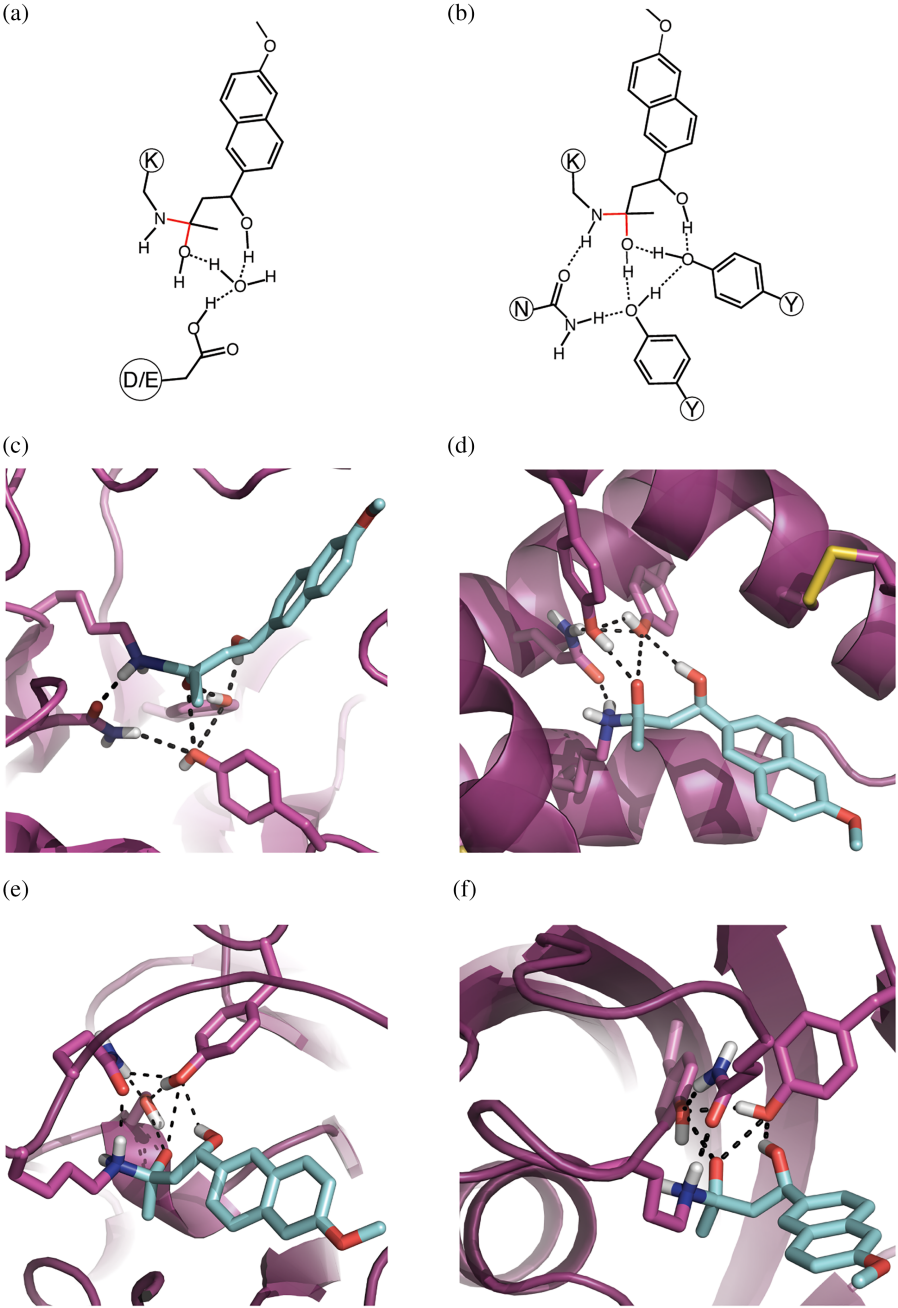
Comparison of the RA95.0 and RA95.5‐8F active sites suggests the importance of catalytic residue connectivity. (a) A two dimensional representation of the RA95.0 theozyme, which consists of Lys210, Glu53, plus a water molecule. (b) Directed evolution of RA95.0 yielded the 200,000‐fold more active RA95.5‐8F, whose catalytic groups include a repositioned lysine, Lys83, along with Tyr180, Tyr51, and Asn110. The three‐dimensional representation of the crystallographic coordinates of RA95.5‐8F are shown in (c) in the context of the complete scaffold, with the networked residues shown in magenta sticks, the ligand in cyan sticks, and hydrogen bonds indicated with black, dashed lines. Analysis of the crystal structure reveals an extensive network of hydrogen bonds between the catalytic side chains, preorganizing the active site. Three‐dimensional realizations of the RA95.5‐8F‐inspired active site generated by HBNetGen in three different protein scaffolds: (d) a lipid transfer protein (scaffold PDB accession code 1bwo); (e) a thymidine kinase (1w4r); and (f) an FKBP‐like domain (1c9h)

We set out to develop a computational method to directly generate such connected catalytic networks. RosettaMatch starts from a geometric description of the optimal interaction geometries between side‐chain functional groups and the transition state (from QM calculations or chemical intuition); to find fully‐connected active sites it is necessary to allow a wider range of functional‐group–transition‐state geometries. We reasoned that potential losses in activity from less optimal placement of individual functional groups relative to the transition state could be more than compensated by the advantages of catalytic network connectivity. The new method, HBNetGen, starts from a ChemDraw‐like specification of the hydrogen‐bonding and partial‐covalent interactions between side‐chain functional groups and the reaction transition state (rather than the more detailed geometric information in the original RosettaMatch), and between the catalytic side chains themselves (much like textbook schematic depictions of enzyme active sites). The transition state is placed on a grid, and side‐chain functional groups are placed around the transition state according to the active‐site specification. The grid enables complete enumeration of compatible functional‐group placements at a resolution set by the (user‐specified) grid spacing. At a grid spacing of 0.2 Å, there are typically ~10^3^–10^4^ functional‐group placements for each individual interaction in the active‐site specification. Next, for each side‐chain–side‐chain hydrogen bond in the connected active‐site specification, each pair of functional‐group placements for the two side chains is queried for the presence of the hydrogen bond, and placements which do not make the required interaction with any of the enumerated placements for the second side chain are discarded. Catalytic networks are then completed by joining the pairs of hydrogen‐bonding residues, and fully‐connected networks which make all the specified interactions between catalytic side chains and transition state and between the catalytic side chains are output.

To test HBNetGen, we selected seven examples of fully‐connected catalytic networks in the PDB and ran HBNetGen on these crystal structures and that of the evolved retroaldolase, starting from the backbone coordinates and a ChemDraw description of the connectivity of the desired active site. For direct comparison with the crystal structures, the inhibitor or reaction product was used in these tests rather than the computed reaction transition state. The results are summarized in Figure 2: for each ligand, the left‐most panel shows the two‐dimensional description of the network as a ChemDraw image, the center panel shows an example of a complete network at the functional‐group level with the number of generated networks indicated below, and the right‐most panel shows a full‐side‐chain representation of the network with the number of unique backbone positions for the selected side‐chain identities indicated below. The number of fully‐connected network solutions ranges from 7,267 for the alanine phosphonate network to 1,534,996 for the retroaldolase network.

**Figure 2:**
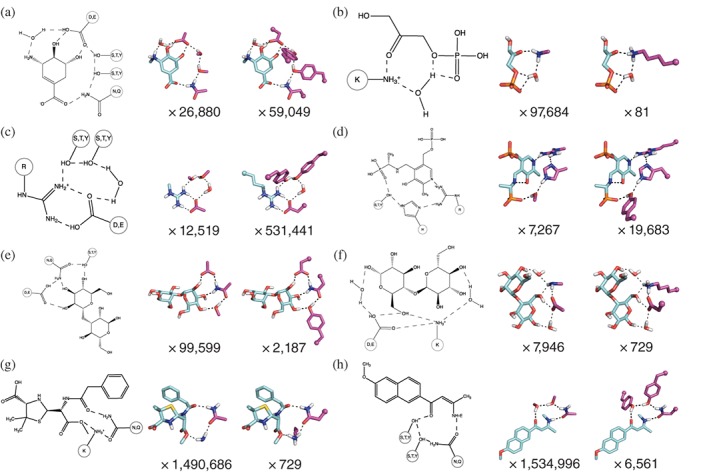
Generation of HBNetGen three‐dimensional (3D) active sites from two‐dimensional (2D) ChemDraw wiring diagrams. Eight candidate networks surrounding ligands were selected from the PDB: (a) (4R,5R)‐3‐amino‐4,5‐di‐ hydroxy‐cyclohexene‐1‐carboxylate; (b) 1,3‐dihydroxyacetone‐phosphate; (c) arginine; (d) {1‐[(3‐hydroxy‐methyl‐5‐phosphonooxy‐methyl‐pyridin‐4‐ylmethyl)‐amino]‐ethyl}‐phosphonic acid; (e) 3‐O‐ɑ‐D‐mannopyranosyl‐ɑ‐D‐mannopyranose; (f) maltose; (g) the open form of penicillin G; and (h) the retro‐aldol intermediate found in RA95.5‐8F. Each row shows: (left) a 2D representation of the interactions that form a complete network; (middle) the lowest‐energy network configuration at the functional‐group level, with the total number of network configurations indicated below; and (right) a full side‐chain representation of a network configuration, with total number of full side‐chain realizations for the network configuration (dependent on the number of rotatable bonds for the constituent side chains) indicated below. The total number of full side‐chain 3D realizations of the 2D connected reaction schematic on the left is the production of the numbers in the second and third columns: (number of 3D placements of functional groups) × (number of sidechain placements per functional group placement)

To identify placements of the connected networks in sets of protein scaffolds, we adapted the previously described RosettaMatch method to input the rigid‐body transformations for each residue in each network expressed in a hierarchical XML file: a RosettaMatch calculation using the side‐chain conformations from a single HBNetGen network will by construction only identify fully‐connected active sites. However, even with the efficiency of RosettaMatch, searching for placements of hundreds of thousands of designed, connected active sites in protein scaffolds, each with a unique set of side‐chain conformations, is not computationally feasible for any but the simplest active sites. To make this problem tractable, we experimented with pooling the side‐chain conformations from all HBNetGen networks, and then using RosettaMatch to find combinations of side chains making the specified interactions with the transition state. This solution has the advantage of ensuring that each rotamer at each position is only considered once during the calculation, which considerably increases computational efficiency, but has the consequence that side‐chain conformations from different networks can be mixed during matching. We speculated that side‐chain rotamer conformations in the same HBNetGen network would have a higher likelihood of being placed in the same active site by RosettaMatch because of their geometric complementarity, leading to recovery of many of the input networks. However, fully‐connected networks were recovered infrequently with this approach (see next paragraph). To modulate the extent of network remixing, we experimented with clustering the set of networks on the coordinates of the central functional groups; this allows smooth interpolation between the case where each network is treated independently (cluster size 1), and that where all networks are combined (one large cluster). A larger number of smaller clusters will produce fewer models with incomplete networks but will increase the runtime because a separate calculation must be performed for each cluster; we found that using ~250 clusters balanced performance and the run‐time increase.

To evaluate the performance of HBNetGen‐guided match generation, we matched the set of networks inspired by the evolved retro‐aldolase (RA95.5‐8F) active site, with the additional criterion that each side chain must interact with the ligand directly (Figure S3), into a set of ~6,000 ligand‐binding scaffolds.^20^ We modeled the transition state for carbinolamine formation with a partial covalent bond to the nucleophilic lysine, and generated positions of the remaining three residues using HBNetGen, which produced 4,858 networks. Clustering produced 222 clusters comprising 4,526 networks. We compared three approaches: (a) the original RosettaMatch residue‐based method that considers each residue completely independently using functional group geometries based solely on interaction geometry with the substrate; (b) RosettaMatch using all 4,858 sidechain conformations from all networks identified by HBNetGen in one simulation; and (c) independent RosettaMatch calculations using side‐chain conformations from each of 239 clusters of HBNetGen networks (thus retaining the primary side‐chain dependencies determined by HBNetGen). For each resulting active site placement, we determined whether the desired connectivity of the catalytic side chains was achieved. The third approach (using the 239 networks resulting from clustering) generated 98 fully‐connected active site solutions from 3,031 matches, the second approach (using all side chain arrangements from all 4,858 networks) yielded 3,991 matches, but fewer (47) connected networks, and control RosettaMatch calculations identified only one fully‐connected network (Table 1; in all cases, most of the matches involve only partial networks, as expected because RosettaMatch considers each residue individually in order to make the search computationally tractable). The smaller number of fully‐connected networks found in the second approach compared to the third approach is due to pruning within RosettaMatch to reduce the combinatorial complexity of the sampling problem (see Supporting Information). Figure 1d–f shows fully‐connected matches of the RA95.5‐8F‐inspired theozyme in three different scaffolds. The networked residues are shown in magenta sticks, the ligand is shown in cyan sticks, and hydrogen bonds are indicated with black, dashed lines. While all the matches have the connectivity of the input two‐dimensional active site depiction, the three‐dimensional realizations are quite different.

**Table 1:** Placement of HBNetGen‐designed RA95.5‐8F inspired catalytic networks into protein scaffolds

Simulation ID	Number of incomplete networks	Number of complete networks	Completion rate
Residue‐based^a^	7,455	1	0.013%
Network‐based^b^ (all)	3,991	47	1.18%
Network‐based^b^ (clustered)	3,031	98	3.23%

a Side‐chain geometries from rigid‐body transformation to ligand for each residue separately (default RosettaMatch settings).

b Side‐chain geometries from networks produced by HBNetGen, tested with, and without clustering.

HBNetGen explicitly generates fully‐connected active sites that are likely to be more preorganized than previous *de novo* designed catalytic sites. It allows exploration of different catalytic‐site specifications (at the ChemDraw level), completely independent of a particular protein backbone. This capability enables determination of the extent to which different sites can be realized in three dimensions with full hydrogen bonded connectivity, and investigation, again independent of any protein backbone, of whether the active site configurations found in nature were favored because of the transition state stabilization they provide or because of the connectivity of the catalytic side chains. It is likely that algorithms for finding matches to the HBNetGen connected sites in actual protein structures can be developed that are more efficient than the simple RosettaMatch implementation described here which breaks up the networks for computational tractability. Experimental characterization of HBNetGen fully‐connected active sites should provide insight into the contribution of preorganization and side‐chain connectivity to catalysis.

## CONFLICT OF INTEREST

The authors declare no competing financial interest.

## Supporting information


**Appendix** **S1** Supporting Information
