# Supplementary material for: A computational method for design of connected catalytic networks in proteins
Source: Protein Sci. 2019 Nov 19;28(12):2036–41. doi: 10.1002/pro.3757 (PMC6863703; doi:10.1002/pro.3757)
Supplement: Supplementary file 1 — Appendix S1 Supporting Information [file PRO-28-2036-s001.docx]

Computational method for design of connected catalytic networks in proteins – supporting information

Brian D. Weitzner^1,2,†,‡^, Yakov Kipnis^1,2,‡^, A. Gerard Daniel^1,2,‡^, Donald Hilvert^3^, David Baker^1,2,4,*^

^1^Department of Biochemistry, University of Washington, Seattle, WA 98195, USA.

^2^Institute for Protein Design, University of Washington, Seattle, WA 98195, USA.

^3^Laboratory of Organic Chemistry, ETH Zurich, 8093 Zurich, Switzerland.

^4^Howard Hughes Medical Institute, University of Washington, Seattle, WA 98195, USA

Supporting Information

Methods

HBNetGen algorithm

HBNetGen is a C++ application built to solve the problem of generating three-dimensional structures of fully-connected hydrogen bond networks from a two-dimensional description of an active site, such as a ChemDraw diagram. The program requires a Rosetta-compatible description of the ligand (see <https://www.rosettacommons.org/demos/latest/tutorials/prepare_ligand/prepare_ligand_tutorial> for instructions) and the set of atoms (using the names in the params file) on the ligand, termed anchor atoms, paired with the one-letter codes of user-specified side chains for each interaction as input. Each ligand conformation must be considered in a separate simulation because the hydrogen bond network’s connectivity can depend on the conformation of the ligand. For consistency, the ligand’s conformation is not sampled during the subsequent RosettaMatch phase. As shown in Figure S1, HBNetGen consists of four stages: (1) node sampling; (2) binary link detection; (3) network completion and (4) residue stub building and filtering. Each of these stages is discussed below.

Node sampling

A coordinate frame is defined around each anchor atom specified. The anchor atoms are taken as the root nodes of separate trees. Discrete positions for the hydrogen bonding atom complementary to the anchor atom type are placed on a grid around the anchor atom using the reference coordinate frame. The grid spacing (resolution) and an initial distance from the ligand atom can be adjusted by the user with command line arguments at run time. Atoms that do no clash with any ligand atoms are stored as children of the root node. Child nodes contain the position, atom type and its relationship to the parent. For instance, if the anchor atom is an 'Acceptor' type, there would be a spherical grid (minus points that clash with the ligand) of nodes of type 'Hydrogen' related to the parent or root node by 'H-bond'.

Reference coordinate frames are then defined for each child node in order to allow them to act as the parent common to any residue that consists the node’s atom type. To illustrate, continuing from the previous example, the nodes of type 'Hydrogen' and relation to parent 'H-bond' can be the parent of amino acids that have a polar hydrogen (Arg, Asp, Ser, etc.) or water. Parameters derived from structural data are used to sample these second-level nodes, which now represent bonded polar atoms of the appropriate residue type. For residues with more than two polar atoms, we perform a third level of sampling. Third-level sampling is simplified by virtue of having two of the other atom positions defined, reducing the sampling degrees of freedom to two. Clash checking against ligand atoms is performed at each level of sampling; only clash-free positions are saved as nodes. Second shell or bridging residues are sampled using the same procedure with a larger initial distance parameter.

Binary link detection

At this stage, we have constructed a tree of all polar-atom positions for each of the user-specified interactions rooted at their respective ligand anchor atoms. Our eventual task is to find fully-connected networks of residues, so we first search for binary links between each of the trees. Pairs of trees are traversed, and each pair of polar atoms’ positions are used to determine whether they are within hydrogen bonding distance. If so, these are saved as binary links.

Selecting the pairs of trees to search can have a significant impact on runtime, as the number of possible binary links expands. As such, the search can consider every possible pair of trees or only pairs that are relevant to the network as specified by the user. In addition to affecting runtime, the choice of tree pairs can be a critical step that determines the connections in the final network.

Network completion

Once binary links are identified, they must be assembled into complete networks. This is accomplished by searching for pairs of binary links that share nodes from the same tree or hydrogen bonding parent. For instance, consider the trees be represented by the indices {1, 2, 3, 4}. Let a binary link {(1, A), (2, B)} exist such that nodes A and B belong to trees 1 and 2, respectively. If there exists another binary link {(1, C), (3, D)}, this pair of links would extend the network because tree 1 is present in both links.

In the case of three-residue networks, assembling two binary links results in a complete network. However, in cases in which there are more than three anchors specified, combining two binary links produces a partially connected network. In these cases, the process is repeated searching for binary links to extend the partial networks and complete the network.

Residue stub building and filtering

Given a set of complete networks consisting of hydrogen-bond forming atoms, we place the remaining side-chain atoms, sampling χ angles when appropriate. Conformations free of steric clashes are collected and written to disk in a PDB-formatted file with TER record separating each network.

Updates to the Matcher

For a desired network topology, HBNetGen can generate tens- or hundreds-of-thousands of fully-connected networks. This presents the challenge of considering all networks when placing residues into a particular scaffold, which we address through the creation of a new XML-based constraint file format.

The classic constraint-file format used by the Matcher consists of blocks describing each interaction between a residue and the ligand. The block defines constraints as a rigid-body transformation expressed as a distance, two angles, and three dihedral angles between coordinate frames computed by the Cartesian coordinates of three atoms on the residue and three atoms the ligand. Each parameter in the constraint supports optional additional sampling by adding more sample points at even intervals within a user-specified tolerance. While this allows for increased sampling, additional points must be sampled regardless of whether those conformations are consistent with the desired result.

To account for this, we compute the rigid-body transformations for each residue in each network and express them in hierarchical XML file. Each interaction is defined in a MatcherConstraint tag that can have any number of rigid-body transformations each in a separate Combination subtag. The Combination tags include all of the information that was available in the classic file format.

We modified the Rosetta source code to be able to validate the XML-based constraints at load time, which ensures that it is properly formatted before the start of the simulation. We then added the ability to read the XML files and construct vectors of sample points based on the Combination tags to the Matcher. When the Matcher iterates over each rotamer of a residue in an encoded interaction, it also iterates over each sample point in specified in the Combination tags and keeps track of the Combinations that result in hits. This solution has the advantage of ensuring that each rotamer at each position is only considered once during the simulation, allowing the simulation to proceed quickly while preserving the strengths of the Matcher. The Matcher breaks the network in order to allow each interaction to be considered independently, which makes the problem computationally tractable but has the consequence of allowing network components to be re-mixed during matching. However, the current implementation of the Matcher only retains one conformation for each hashing bin, which causes rotamers that position the ligand identically to a to be pruned to a single rotamer. Because of this, results from the Matcher are not guaranteed to contain all of the interactions specified in the network. Future development efforts on RosettaMatch could include an adaptation to retain all rotamers that lead to identical substrate placements to improve network recovery.

Benchmark run

Theozyme geometry generation

For benchmarking, we constructed a theozyme representative of the transition state of the nucleophilic attack catalyzed by RA95.5-8F (retro-aldolase) on its substrate, methodol (2-Butanone,4-hydroxy-4-(6-methoxy-2-naphthalenyl)-). We refer to each of the theozyme residues (corresponding to K83, N110, Y51, Y180 in RA95.5-8F) as “nucleophile” (“nuc”), “bridge” (“brd”), “shuttle” (“sht”) and “support” (“sup”), respectively. The transition state model includes two stereocenters, so we constructed one model for each stereoisomer (four models total) to run independently within the Matcher. Additionally, residues with identical functional groups were allowed in addition to the native residue. That is, Gln and Asn were allowed in the “bridge” position, and Ser, Thr, and Tyr were allowed as the “support” residue.

The classic matcher constraint file format can be used to describe the rigid-body transformations within the theozyme to place the “nucleophile”, “shuttle” and “support” residues relative to ligand by applying classical matching algorithm, as well as the placement of the “bridge” residue relative to already-determined locations of the “nucleophile” using the secondary matching method. Limitations of the legacy Matcher implementation do not allow descriptions of the remaining three interactions to be considered. In this configuration, one constraint file corresponds to a single network.

We described the hydrogen bonding interactions of the RA95.5-8F active site with a tetrahedral reaction intermediate and generated coordinates for all connected networks using HBNetGen. Next, we used the coordinates of the hydrogen bonding atoms and two other atoms in the side chain to produce the six geometric parameters (distance, two angles, three dihedral angles) defining the rigid-body transformation from a set of three arbitrarily-chosen ligand atoms for each residue in each network. We then formatted these parameters into an XML-style constraint file used for matching.

Scaffold set construction

For the benchmark, a set of nearly 6000 ligand-binding proteins was constructed using the BindingMOAD database. Each structure was processed by splitting it into individual chains, followed by relaxation with coordinate constraints to identify high-energy features according to the Rosetta Energy Function, determination of the positions of amino acids comprising the protein–ligand binding site, as well as removal of all non-protein atoms and non-canonical amino acids.^1^

Post-matching optimization

We used the RosettaScripts interface to perform matching with either the classic matcher constraint format or XML-style constraints derived from HBNetGen produced networked theozymes.

After matching, we optimized all of the models to account for any effects of the discrete rotamer-sampling and ligand-position binning used by the Matcher. Optimization proceded in the following four steps: (1) all non-theozyme residues are converted to Ala to open the active site; (2) coordinate constraints are applied to the side-chain heavy atoms of the theozyme residues to restrict movement; (3) distance and angle constraints are applied to hydrogen bonding atoms; and (4) the coordinates of the protein are updated by minimization with respect to the full-atom Rosetta Energy Function. After optimization, models with theozyme-residue hydrogen bonds scoring better than -0.5 kCal/mol are outputted for further processing.

Anchor points and network topologies.

HBNetGen requires sensible definition of the network used as an input. It is not uncommon for enzyme active sites to change pattern of H-bond connectivity (topology of the network), progressing through reaction cycle, without substantial changes in position of heavy atoms.

It is important to get anticipated network topology as accurate as possible before submitting the computation, since there is no currently implemented heuristic, responsible for the analysis of the topological incompatibilities or ambiguities in the input.

In its current form HBNetGen cannot automatically sample network topologies when sp3 protons are used as anchors. In the figure S2 networks in the quadrants I-IV will be produced if hydroxyl oxygen is used as an anchor of the magenta branch of the network. Ignoring case of the trivial network I, where connection between magenta colored and blue colored branches of the network is formed by intraligand H-bond (heavy dashed line), it may be desired to bias the search for networks towards topology similar to the one in III. Setting hydrogen as an anchor point for the magenta branch to achieve that (see lower panel of S3 figure for postulated network of the RA95.5-8F as an example) will require external sampling of the anchor position as it is not currently implemented in HBNetGen.

Grouping networks for matching by clustering on theozyme components

RosettaMatch can be run on each individual network produced by HBNetGen and individual scaffold of interest. This computation takes approximately a minute and if successful, accurately recapitulates original network in the protein scaffold (minor deviations from the original geometry are possible due to inherent tolerances of the Matcher algorithm). However, this computational setup is associated with substantial time spent on preparatory steps such as reading input files and necessary databases. Alternatively, it is possible to combine geometries of all networks in one xml constraint file to search for matches in given protein scaffold with minimal computational overhead to initiate RosettaMatch run. Combining all networks leads to loss of information about each individual network and promotes recombination of geometrical parameters for individual interactions originating from different networks. This results in solutions overwhelmingly dominated by incomplete networks and makes it hard to trace origin of each result to a particular network. To speed up computations without getting excessive recombination we clustered networks based on position of one of the theozyme components, generated xml constraints for each cluster and used them for matching. Clustering on position of the “shuttle” component of the RA theozyme resulted in substantial decrease in number of incomplete networks in RosettaMatch output.

Availability

We have collected a set of scripts and instructions to replicate the workflow described in this manuscript and made them available in a public GitHub repository hosted at https://github.com/weitzner/HBNetGen_example

References

1. Nivon LG, Moretti R, Baker D. A Pareto-optimal refinement method for protein design scaffolds. PLoS One 2013;8(4):e59004.

**Supplementary Figures**

**Figure S1:** Overview of HBNetGen routine. The HBNetGen routine consists of four steps. (1) Node sampling: the user-specified anchor atoms are used as the root nodes of separate trees. Discrete positions for complementary hydrogen-bonding atoms are placed on a grid around the anchor atom as nodes, and this process is repeated until all polar side-atoms have been placed. Only trees that do not clash with the ligand are retained. (2) Binary link detection: Pairs of trees are saved as binary links if they can form a hydrogen bond between them. (3) Network completion: Pairs of binary links that share nodes from the same tree or hydrogen bonding parent are merged and referred to as networks. This process is repeated, and networks that visit every anchor atom on the ligand are considered complete. (4) Residue stub building: Remaining side-chain atoms are placed, sampling χ angles when appropriate, on the complete networks. Clash-free conformations free are collected and written to disk.


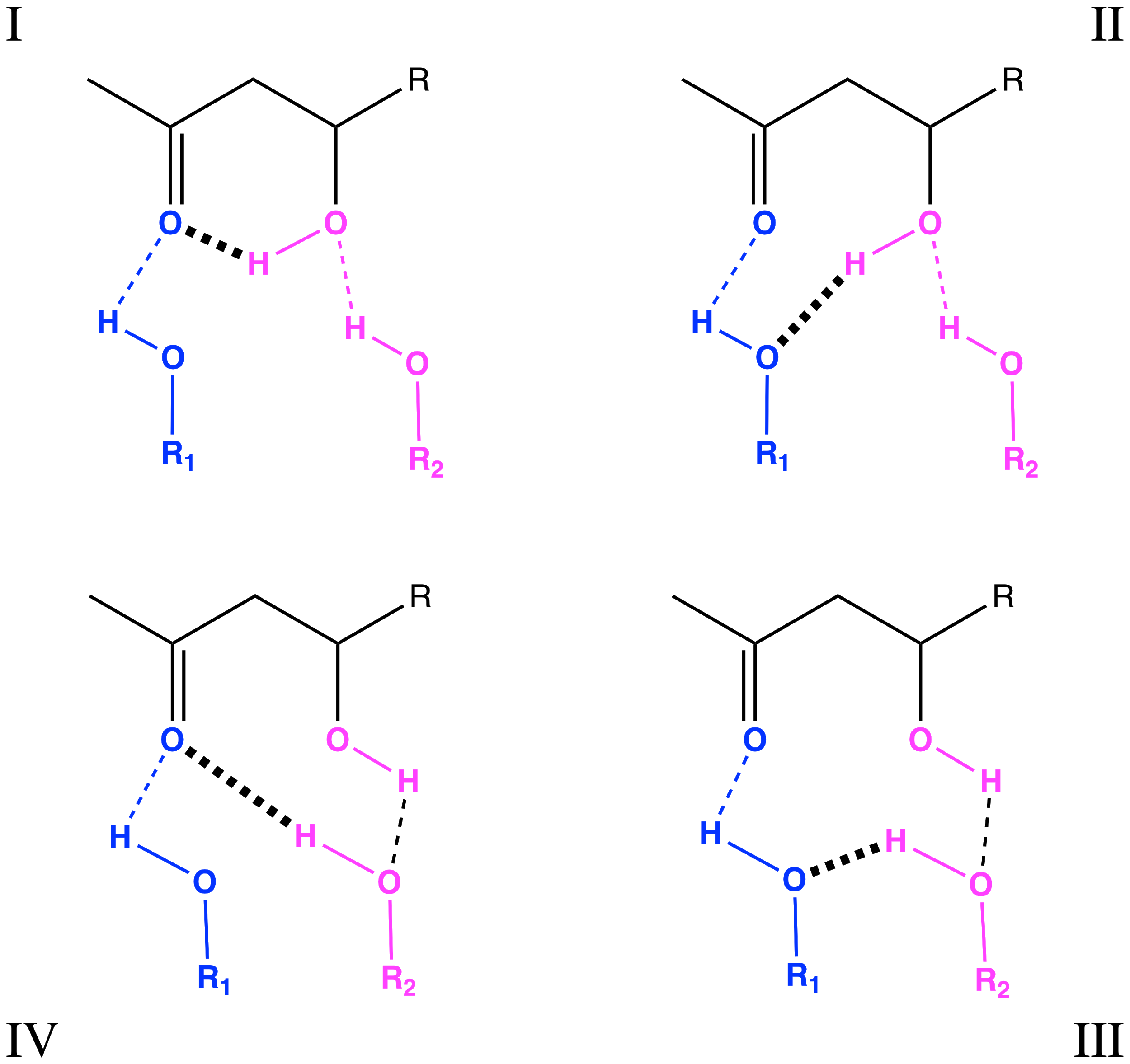


**Figure S2:** Different network topologies involving the same residues. The networks in quadrants I-IV will be produced if hydroxyl oxygen is used as an anchor of the magenta branch of the network. Ignoring case of the trivial network I, where connection between magenta colored and blue colored branches of the network is formed by intraligand H-bond (heavy dashed line), it may be desired to bias the search for networks towards topology similar to the one in III. Setting hydrogen as an anchor point for the magenta branch to achieve that will requires external sampling of the anchor positions in the ligand as it is not currently implemented in HBNetGen.


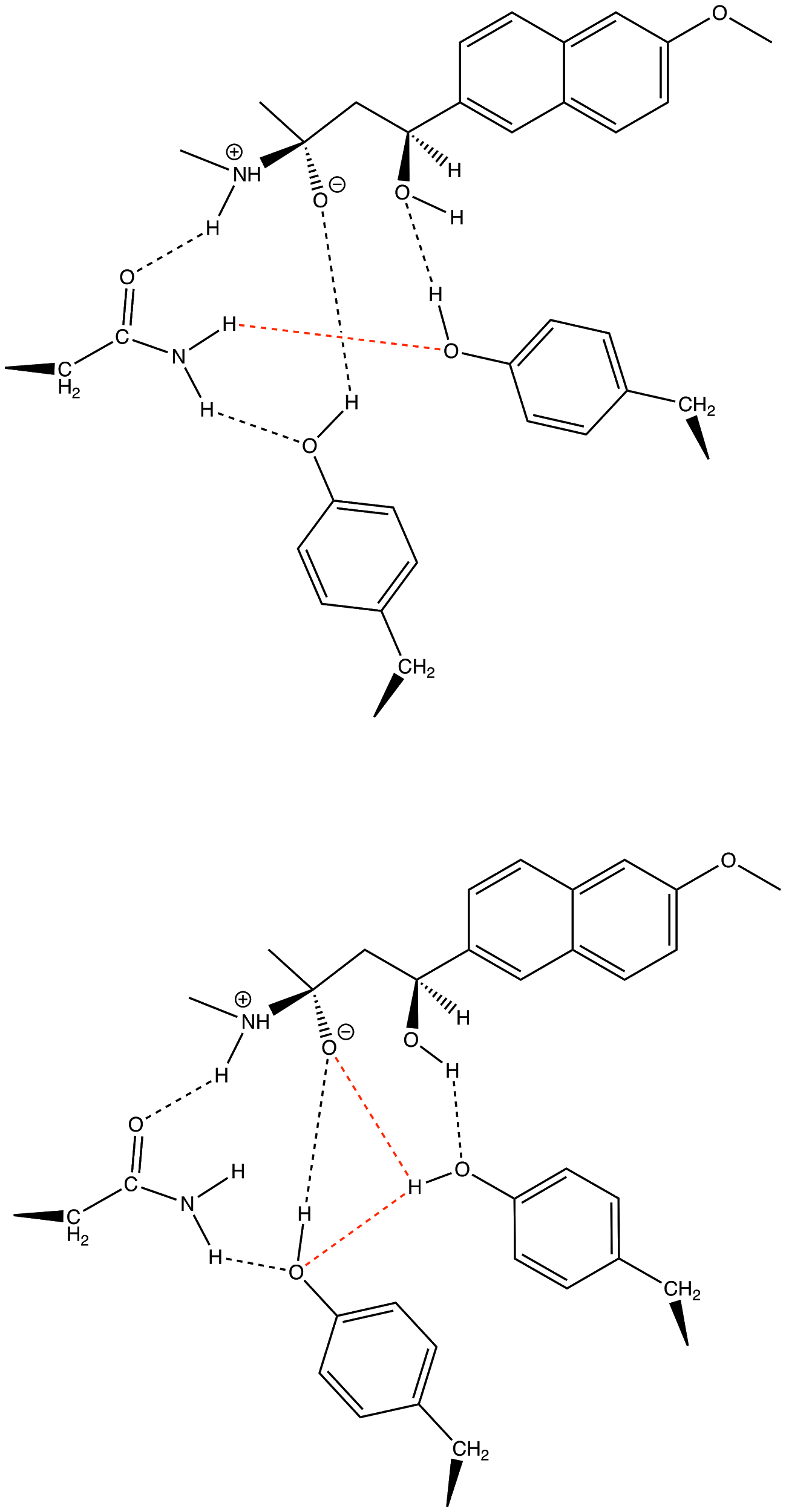


**Figure S3:** Postulated networks of RA95.5-8F. Two different connectivities of the network in RA95.5-8F are shown. The top network allows the tyrosine shown at the bottom of the panel more freedom, but may have less of a pre-organizing effect. The network shown in the bottom panel is more restricted, and likely serves to organize the active site more strongly than the network shown in the top panel. The network shown in the bottom panel was used in this work.

Flag file used to set up network generation of RA95.5-8F theozyme

-database /work/geradda/Rosetta/database

-polar_network_generation

-ligand_position 1

-HB_anchor_atoms_and_AAs O2:Y O3:Y 2HZ:N

-HB_dist_min 1.8

-HB_dist_max 2.0

-HB_dist_step 0.2

-HB_grid_resolution 2

-inter_res_HB_dist_min 1.8

-inter_res_HB_dist_max 2.2

-sec_HB_anchor_atoms_and_AAs

-sec_HB_dist_min 2.4

-sec_HB_dist_max 2.4

-sec_HB_dist_step 0.2

-sample_proton_chis_at_atoms O2

-sample_AAs_at_chi_protons true

-catalytic_residue_is_at C11:K

-residue_atoms_sampling_resolution 2

-proton_sampling_level 2

-sidechain_chi_sampling_level 2

-KR_chi_sampling_level 1

-sample_catalytic_residue_terminal_chi false

-treat_intraligand_HB_as_link false

-clash_cutoff_distances 2.6:1.7:2.2:2.4:2.6:2.8:3.2:3.4

-output_networks_for_docking_later true

-include_ligand_polar_anchor_atoms_in_network true

-include_ligand_in_output true

-user_defined_polar_atoms_and_types

Network clustering script

from collections import namedtuple

import attr

import logging

import numpy as np

import os

logging.basicConfig(level=logging.INFO)

@attr.s

class AtomRecord(object):

record_name = attr.ib()

serial = attr.ib()

name = attr.ib()

altLoc = attr.ib()

resName = attr.ib()

chainID = attr.ib()

resSeq = attr.ib()

iCode = attr.ib()

x = attr.ib()

y = attr.ib()

z = attr.ib()

occupancy = attr.ib()

tempFactor = attr.ib()

element = attr.ib()

charge = attr.ib()

@classmethod

def from_str(cls, record):

record_name = record[:6].strip()

serial = int(record[6:11].strip())

name = record[12:16].strip()

altLoc = record[16].strip()

resName = record[17:20].strip()

chainID = record[21].strip()

resSeq = int(record[22:26].strip())

iCode = record[26].strip()

x = float(record[30:38].strip())

y = float(record[38:46].strip())

z = float(record[46:54].strip())

occupancy, tempFactor, element, charge = [None] * 4

"""

try:

occupancy = float(record[54:60].strip())

tempFactor = float(record[60:66].strip())

element = record[76:78].strip()

charge = record[78:80].strip()

except (IndexError, ValueError):

pass

"""

return cls(

record_name,

serial,

name,

altLoc,

resName,

chainID,

resSeq,

iCode,

x,

y,

z,

occupancy,

tempFactor,

element,

charge,

)

def __str__(self):

return "{record_name:6}{serial:5d} {name:4}{altLoc:1}{resName:3} {chainID:1}{resSeq:>4}{iCode:1} {x:8.3f}{y:8.3f}{z:8.3f}{occupancy:6.2}{tempFactor:6.2} {element:2}{charge:2}".format(

record_name=self.record_name,

serial=self.serial,

name=self.name,

altLoc=self.altLoc,

resName=self.resName,

chainID=self.chainID,

resSeq=self.resSeq,

iCode=self.iCode,

x=self.x,

y=self.y,

z=self.z,

occupancy=self.occupancy if self.occupancy is not None else "",

tempFactor=self.tempFactor if self.tempFactor is not None else "",

element=self.element if self.element is not None else "",

charge=self.charge if self.charge is not None else "",

)

@attr.s

class Residue(object):

atom_records = attr.ib()

coords = attr.ib()

@classmethod

def from_records(cls, atom_records):

coords = np.array([[ar.x, ar.y, ar.z] for ar in atom_records])

return cls(atom_records, coords)

@property

def name(self):

names = set(ar.resName for ar in self.atom_records)

assert(len(names) == 1)

return names.pop()

def __str__(self):

return "\n".join([str(ar) for ar in self.atom_records])

def read_in_stubs_file(fname):

models = []

model = []

curr_res = []

resSeq = 0

with open(fname, "r") as f:

for l in f:

if not l:

continue

if l.startswith("MODEL"):

# clear previous model data

model = []

curr_res = []

resSeq = 0

continue

if l.startswith("ENDMDL"):

# finalize current model

model.append(Residue.from_records(curr_res))

models.append(model)

continue

record = AtomRecord.from_str(l)

if record.resSeq == resSeq:

curr_res.append(record)

else:

if curr_res:

model.append(Residue.from_records(curr_res))

curr_res = [record]

resSeq = record.resSeq

return models

def process_stubs(fname, n_clusters=100):

base_name, ext = os.path.splitext(fname)

if not os.path.exists(base_name):

os.makedirs(base_name)

logging.info("Reading in file...")

# filter out silly broken models from the list as soon as they are returned

models = [mdl for mdl in read_in_stubs_file(fname) if len(mdl) == 5]

logging.info("File processed!")

logging.info("Extracting SER coords...")

ser_coords = np.array([mdl[1].coords for mdl in models])

logging.info("SER coords exrtracted!")

logging.info("Extracting TYR coords...")

tyr_coords = np.array([mdl[2].coords for mdl in models])

logging.info("TYR coords exrtracted!")

logging.info("Combining...")

res_coords = np.concatenate((ser_coords, tyr_coords), axis=1)

# flatten array so each residue is one coordinate in a higher-dimensional space

logging.info("Resizing coord array for clustering...")

res_coords = res_coords.reshape(

res_coords.shape[:-2] + (res_coords.shape[-1] * res_coords.shape[-2],)

)

logging.info("Done!")

# cluster on the TYR coordinates

logging.info("clustering...")

from sklearn.cluster import DBSCAN

clustering = DBSCAN(eps=0.25, metric="euclidean", n_jobs=-1).fit(res_coords)

logging.info("Done!")

from itertools import groupby

# identify top N clusters

top_n_clusters = sorted(

[(key, len(list(group))) for key, group in groupby(sorted(clustering.labels_))],

key=lambda x: x[-1],

reverse=True,

)[:n_clusters]

# "noisy" samples are given the label -1, so if -1 is included in the top N,

# we should remove it

top_n_clusters = [cluster for cluster in top_n_clusters if cluster[0] >= 0]

# collect groups of model IDs for the top N clusters

# ensure they are consistent with the previous step

ntwrks = []

for clusID, n_occ in top_n_clusters:

model_indices_for_network = [

i for i, elem in enumerate(clustering.labels_) if elem == clusID

]

assert len(model_indices_for_network) == n_occ

ntwrks.append(model_indices_for_network)

# write each network cluster to a separate PDB-formatted file

for i, ntwrk in enumerate(ntwrks):

with open(os.path.join(base_name,

"grp_ntwrk_{}_{:04d}".format(base_name, i) +ext), "w") as f:

f.write("MODEL\n")

f.write(

"\nENDMDL\nMODEL\n".join(

["\n".join([str(rsd) for rsd in models[mdlNo]]) for mdlNo in ntwrk]

)

)

f.write("\nENDMDL")

n_clusters = 250

[process_stubs(fn, n_clusters) for fn in os.listdir() if fn.endswith(".pdb")]

count_hbonds.xml

<ROSETTASCRIPTS>

<SCOREFXNS>

<ScoreFunction name="SC_HB_ONLY" weights="empty" >

<Reweight scoretype="hbond_sc" weight="1.0" />

</ScoreFunction>

<ScoreFunction name="BETA" weights="beta_nov16_cart" >

<Reweight scoretype="atom_pair_constraint" weight="0.5" />

<Reweight scoretype="angle_constraint" weight="0.5" />

<Reweight scoretype="dihedral_constraint" weight="0.5" />

<Reweight scoretype="hbond_sc" weight="0.5" />

<Reweight scoretype="coordinate_constraint" weight="0.5" />

</ScoreFunction>

</SCOREFXNS>

<RESIDUE_SELECTORS>

<Index name="lig" resnums="%%lig_resid%%" />

<Index name="nuc" resnums="%%nuc_resid%%" />

<Index name="brd" resnums="%%brd_resid%%" />

<Index name="sht" resnums="%%sht_resid%%" />

<Index name="sup" resnums="%%sup_resid%%" />

<Or name="catres" selectors="nuc,brd,sht,sup" />

<Not name="not_catres" selector="catres" />

</RESIDUE_SELECTORS>

<TASKOPERATIONS>

<OperateOnResidueSubset name="not_catres_to_ALA" selector="not_catres" >

<RestrictAbsentCanonicalAASRLT aas="A"/>

</OperateOnResidueSubset>

<OperateOnResidueSubset name="prevent_catres_to_ALA_and_fix" selector="catres" >

<PreventRepackingRLT/>

</OperateOnResidueSubset>

</TASKOPERATIONS>

<MOVERS>

<DeclareBond name="connect_LYM_LG2" res1="%%nuc_resid%%" res2="%%lig_resid%%" atom1="NZ" atom2="C12" />

<ConstraintSetMover name="add_LYM_cst" add_constraints="1" cst_file="theozyme.cst.txt" /> add_constraints="0" replaces previous csts

<AddConstraints name="add_theozyme_coord_cst" >

<CoordinateConstraintGenerator name="theozyme_coord_cst" residue_selector="theozyme" sidechain="1" sd="0.75" />

</AddConstraints>

<RemoveConstraints name="rm_theozyme_coord_cst" constraint_generators="theozyme_coord_cst" />

<PackRotamersMover name="design_not_catres_to_ALA" scorefxn="BETA" task_operations="prevent_catres_to_ALA_and_fix,not_catres_to_ALA"/>

<MinMover name="min_sc" scorefxn="BETA" bb="0" chi="1" jump="all" cartesian="True" bondangle="True" bondlength="True" type="lbfgs_armijo_nonmonotone" tolerance="0.0001" max_iter="2000" >

<MoveMap>

<Chain number="2" bb="0" chi="0" />

</MoveMap>

</MinMover>

</MOVERS>

<FILTERS>

<HbondsToResidue name="filt_HB_nuc_brd" scorefxn="SC_HB_ONLY" partners="1" energy_cutoff="0.0" backbone="0" bb_bb="0" sidechain="1" residue="%%nuc_resid%%" from_other_chains="0" from_same_chain="1" residue_selector="brd" confidence="0" />HB_nuc_partners

<HbondsToResidue name="filt_HB_brd_sup" scorefxn="SC_HB_ONLY" partners="1" energy_cutoff="0.0" backbone="0" bb_bb="0" sidechain="1" residue="%%brd_resid%%" from_other_chains="0" from_same_chain="1" residue_selector="sup" confidence="0" />HB_brd_partners

<HbondsToResidue name="filt_HB_sht_lig" scorefxn="SC_HB_ONLY" partners="2" energy_cutoff="0.0" backbone="0" bb_bb="0" sidechain="1" residue="%%sht_resid%%" from_other_chains="1" from_same_chain="0" residue_selector="lig" confidence="0" />HB_sht_partners

<HbondsToResidue name="filt_HB_sht_sup" scorefxn="SC_HB_ONLY" partners="1" energy_cutoff="0.0" backbone="0" bb_bb="0" sidechain="1" residue="%%sht_resid%%" from_other_chains="0" from_same_chain="1" residue_selector="sup" confidence="0" />HB_sup_partners

<HbondsToResidue name="filt_HB_sup_lig" scorefxn="SC_HB_ONLY" partners="1" energy_cutoff="0.0" backbone="0" bb_bb="0" sidechain="1" residue="%%sup_resid%%" from_other_chains="1" from_same_chain="0" residue_selector="lig" confidence="0" />HB_sup_partners

</FILTERS>

<PROTOCOLS>

<Add mover_name="connect_LYM_LG2" />

<Add mover_name="add_LYM_cst" />

<Add mover_name="design_not_catres_to_ALA" />

<Add mover_name="add_theozyme_coord_cst" />

<Add mover_name="min_sc" />

<Add mover_name="rm_theozyme_coord_cst" />

<Add filter_name="filt_HB_nuc_brd" />

<Add filter_name="filt_HB_brd_sup" />

<Add filter_name="filt_HB_sht_lig" />

<Add filter_name="filt_HB_sht_sup" />

<Add filter_name="filt_HB_sup_lig" />

</PROTOCOLS>

<OUTPUT scorefxn="SC_HB_ONLY" />

</ROSETTASCRIPTS>
